# Can gastropexy reduce the recurrence rate after paraesophageal hernia repair? A study protocol for a double blind, randomized, multicenter clinical trial

**DOI:** 10.1186/s13063-026-09578-7

**Published:** 2026-03-16

**Authors:** J. Blixt Dackhammar, A. Tsoposidis, V. Wallenius, S. Kostic, Y. Cengiz, G. Linder, M. Jeremiasen, A. Analatos, L. Lundell, B. Håkanson, A. Thorell, M. Reuterwall Hansson

**Affiliations:** 1 Department of Clinical Sciences, Danderyd Hospital, Karolinska Institutet, Stockholm, Sweden; 2 Department of Surgery, Ersta Hospital, Stockholm, Sweden; 3 Department of Surgery, Sundsvall Hospital, Sundsvall, Sweden; 4 Department of Surgery, Sahlgrenska University Hospital, Göteborg, Sweden; 5 Department of Surgical Sciences, Uppsala University, Uppsala, Sweden; 6 Department of Surgery, Skane University Hospital, Lund, Sweden; 7 Division of Surgery and Oncology, CLINTEC, Karolinska Institutet, Stockholm, Sweden

**Keywords:** Paraesophageal hernia, Hiatal hernia repair, Crural repair, Hiatal hernia recurrence, Gastropexy, Nissen fundoplication, GERD, Obstructive symptoms, Quality of life, Patient reported outcome measures

## Abstract

**Background:**

Despite several attempts to improve the durability of the reconstruction of paraesophageal hernias (PEH), recurrence rates remain high. Gastropexy has often been added to the reconstruction with the intention to reduce recurrence rates by anchoring the stomach in the abdominal cavity. The efficacy of adding gastropexy to standard PEH repair has, however, not yet been investigated in a randomized controlled trial setting.

**Methods:**

All patients scheduled for PEH repair are assessed for eligibility for enrolment. Preoperative work-up includes upper GI endoscopy, computed tomography, and symptom assessments. Participants will be randomized (1:1) to either the control or intervention group. In the control group, patients will have a standardized repair, including posterior crural repair and a total fundoplication. In the intervention group, a three-point gastropexy using running non-absorbable sutures will be added to the same reconstruction as in the control group: a posterior fixation of the wrap to the diaphragm, a lateral fixation of the left wrap to the diaphragm, and an anterior fixation of the minor curvature to the inner surface of the anterior abdominal wall. The primary outcome is computed tomography-verified radiological recurrence at 1 year. Secondary outcomes include radiological recurrence at 3 years, patient-reported quality of life, and disease-specific symptoms at 3 months, 1 year, and 3 years, as well as postoperative complications. The study design is double-blinded, with both participants and outcome assessors being blinded to the allocation of patients to treatment arms.

**Discussion:**

Although gastropexy is frequently used in PEH repair with the intent to reduce recurrence rates, the scientific evidence behind its effects is limited. The present trial is the first RCT to evaluate the efficacy of gastropexy combined with a standardized PEH repair.

**Trial registration:**

The trial is registered at ClinicalTrials.gov, NCT06107634. Registered 9 September 2023.

**Supplementary Information:**

The online version contains supplementary material available at 10.1186/s13063-026-09578-7.

## Administrative information

Note: the numbers in curly brackets in this protocol refer to SPIRIT checklist item numbers. The order of the items has been modified to group similar items (see http://www.equator-network.org/reporting-guidelines/spirit-2013-statement-defining-standard-protocol-items-for-clinical-trials/).

**Table:** 

Title {1}	Can gastropexy reduce the recurrence rate after paraesophageal hernia repair? A study protocol for a double blind, randomized, multicenter clinical trial
Trial registration {2a and 2b}	The trial is registered on ClinicalTrials.gov ID NCT06107634.
Protocol version {3}	Version 1.1, 2026–02–04
Funding {4}	ALF-grant from regions Stockholm and Västernorrland The Erling-Persson Foundation The Swedish Medical Society. The Bengt Ihre Foundation
Author details {5a}	Blixt Dackhammar J^1,3^, Tsoposidis, A^4^, Wallenius V^4^, Kostic S^4^, Cengiz Y^3^, Linder G^5^, Jeremiasen M^6^, Analatos A^2^, Lundell L^7^, Håkanson B^1,2^, Thorell A^1,2^, Reuterwall Hansson M^1,2^ ^1^ Department of Clinical Sciences, Danderyd Hospital, Karolinska Institutet, Stockholm, Sweden ^2^ Department of Surgery, Ersta Hospital, Stockholm, Sweden ^3^ Department of Surgery, Sundsvall Hospital, Sundsvall, Sweden ^4^ Department of Surgery, Sahlgrenska University Hospital, Göteborg, Sweden ^5^ Department of Surgical Sciences, Uppsala University, Uppsala, Sweden ^6^ Department of Surgery, Skane University Hospital, Lund, Sweden ^7^ Division of Surgery and Oncology, CLINTEC, Karolinska Institutet, Stockholm, Sweden
Name and contact information for the trial sponsor {5b}	Investigator initiated.
Role of sponsor {5c}	There is no sponsor.

## Introduction

### Background and rationale {6a}

Paraesophageal hernias (PEH) constitute a subtype of hiatal hernias in which large parts of, or the entire stomach, with or without other abdominal viscera, have migrated into the chest cavity through a dilated diaphragmatic hiatus. The prevalence of PEH has been reported to be approximately 3% in middle-aged and older adults, increasing with advancing age [1]. While some PEHs are asymptomatic, others present with a wide range of symptoms including chest or abdominal pain, dysphagia, dyspnea, indigestion, or gastroesophageal reflux (GERD). In severe cases, PEH may also lead to life-threatening complications including volvulus, incarceration, strangulation, or perforation [2–4].

The indication for surgical repair remains controversial. Some suggest that all patients with PEH should undergo surgical repair to avoid acute complications, while many experts argue that only symptomatic hernias should be considered for repair [4–10].

GERD is reported in 30–50% of patients with PEH and can also develop after surgical repair in previously asymptomatic patients. Therefore, the surgical reconstructive procedures should aim not only to restore the anatomy of the diaphragmatic hiatus, remove the hernia sac, and reduce the hiatal opening by adapting the crurae. It should also include an anti-reflux procedure—most commonly a total (Nissen) fundoplication [11–13].

Despite several technical refinements, reported recurrence rates following PEH repair remain high, ranging from 7 to 66% [11, 14–20]. A challenge in PEH repair is that the hiatal reconstruction is exposed to unfavorable physical and functional counteracting forces, promoting both the occurrence and recurrence of PEH, not least due to the significant pressure gradients between the abdominal and thoracic cavities. Coughing, sneezing, vomiting, and heavy lifting are believed to be important factors causing PEH recurrence [21]. Another factor believed to contribute to the high recurrence rates is that the esophagus is permanently attached proximally at the pharyngoesophageal junction and, through its muscular activity, repetitively and forcefully pulls the gastroesophageal junction (GEJ) and the upper part of the stomach in an oral direction.

To prevent recurrence following PEH repair, several surgical techniques have been developed. Among these, procedures in which the stomach is anchored in the abdominal cavity and to the diaphragm by sutures (gastropexy) are among the most commonly practiced [22, 23]. Although the addition of gastropexy to standard PEH repair has garnered widespread clinical attention, evidence supporting its efficacy remains limited [24–28].

### Objectives {7}

The primary objective of this trial is to evaluate if the addition of gastropexy can reduce hiatal hernia recurrence rates at 1 year following PEH repair, compared to crural repair with a total (Nissen) fundoplication alone.

Secondary objectives are to assess if, and if so, how the addition of gastropexy affects the following:

- Recurrence rates at 3 years
- Patient-reported quality of life
- Patient-reported disease-specific symptoms
- Rates and severity of postoperative complications

### Trial design {8}

This study is a randomized, multicenter trial comparing a standardized PEH repair, with or without the addition of gastropexy. The trial design is a parallel group study with block randomization and a 1:1 allocation ratio. The primary objective is to evaluate superiority with the hypothesis that the addition of gastropexy reduces the recurrence rate of radiologically verified hiatal hernia recurrence at 1 year postoperatively.

## Methods: participants, interventions, and outcomes

### Study setting {9}

A nationwide study with six centers in Sweden participating. All procedures will be performed or supervised by an experienced upper gastrointestinal surgeon who has performed more than 25 PEH repairs.

### Eligibility criteria {10}

All individuals aged 18 years or older scheduled for repair of a hiatal hernia grades II–IV (PEH) at participating sites will be considered for entry. Patients will be excluded if they:

- Have undergone previous surgery involving the stomach or the gastroesophageal junction,
- Have achalasia or other major esophageal motility disorders,
- Are unable to understand the purpose of the study and/or are unwilling to give informed consent or
- Have severe comorbidities (defined by the American Society of Anesthesiologists (ASA) physical status score of more than III).

### Who will take informed consent? {26a}

Either a trained research nurse or a surgeon will introduce and explain the purpose and nature of the trial to all patients, providing them with written information about the study. After reviewing the information, patients will have the opportunity to engage in informed discussions with the research nurse or surgeon. Following this discussion, written informed consent will be obtained from all patients who agree to participate in the study.

### Additional consent provisions for collection and use of participant data and biological specimens {26b}

No ancillary studies are currently planned. Any future use of data collected in the present trial for ancillary research will require separate approval from the national ethics review board. Additional participant consent will be obtained if deemed necessary by the ethics authority.

## Interventions

### Explanation for the choice of comparators {6b}

While there are several controversies and no consensus regarding the optimal surgical treatment for paraesophageal hernias, most agree that the standard procedure should include a full mediastinal dissection with adequate esophageal mobilization and complete resection of the hernia sac. In addition, crural repair and an anti-reflux procedure are strongly recommended [6, 8, 9]. The type of fundoplication may affect the risk of postoperative symptoms, such as reflux, bloating, and dysphagia [29, 30]. To ensure standardization, we have chosen to incorporate a total (Nissen) fundoplication, as this technique is the most widely used and recognized worldwide [11].

### Intervention description {11a}

All surgeries will be performed under general anesthesia. The method used for abdominal entry, port placement, and type of liver retractor is at the surgeon’s discretion. Ultrasonic shears are used for dissection. The herniated viscera are completely reduced into the abdomen, and the hernia sac is fully dissected and resected. The esophagus is mobilized intraabdominally until at least 3 cm rests independently below the hiatus without tension. Effort is made to identify and preserve the anterior and posterior vagal nerves. A posterior crural closure with running non-absorbable sutures is performed. An additional anterior crural closure may be performed at the surgeon’s discretion. The fundus is mobilized to allow a floppy fundoplication. A total fundoplication is created by three interrupted non-absorbable sutures [31–33]. No bougies are used routinely for calibration of the fundoplication.

**Fig. 1:**
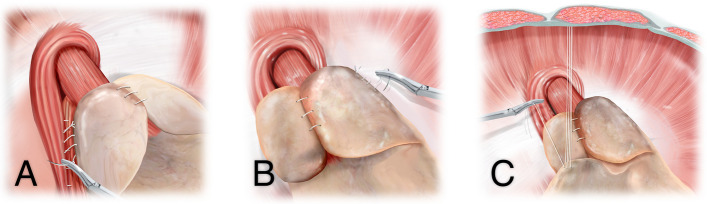
Schematic illustration of the positioning of the gastropexy sutures. (**A**) Posterior gastropexy, (**B**) left lateral gastropexy, and (**C**) anterior gastropexy. The anterior gastropexy is shown before tightening to improve visualization of the planned fixation points

In the intervention group, in addition to the above, a three-point gastropexy is performed (Fig. 1). First, the right fundus flap is adapted posteriorly to the crural portion of the diaphragm with a 2–3 cm long running non-absorbable suture (“posterior gastropexy”). Second, the left fundus flap is adapted to the diaphragm anterolateral to the hiatus with a 2–3 cm long running non-absorbable suture (“left lateral gastropexy”). Finally, the minor curvature of the anterior stomach wall is adapted during reduced intraabdominal pressure to the anterior abdominal wall with a 2–3 cm long running non-absorbable suture (“anterior gastropexy”).

### Criteria for discontinuing or modifying allocated interventions {11b}

If the surgeon deems it necessary to add adjunct procedures or deviate from the protocol in any other way, this is noted in the operative case report form as well as in the medical record.

### Strategies to improve adherence to interventions {11c}

Before the start of the trial, surgical techniques will be standardized and harmonized across the participating sites. A video demonstrating the proper technique for the intervention will be provided to all sites. Each site is required to submit a video illustrating the procedure as performed at the respective center. These videos will be reviewed by the trial steering committee to verify adherence to the standardized surgical techniques before commencement of operating patients within the study, with particular focus on gastropexy techniques, especially anterior gastropexy, including confirmation of anatomical landmarks, fixation points, and suture placement to ensure procedural standardization across centers.

### Relevant concomitant care permitted or prohibited during the trial {11d}

The PEH repair must strictly adhere to the study protocol to ensure consistency across centers. However, there are no restrictions on perioperative or other medical care outside the scope of the protocol.

### Provisions for post-trial care {30}

Healthcare in Sweden is publicly funded. Post-trial care will be provided by the referring or operating hospital. In case of harm, any compensation needed is provided by the Swedish patient insurance system.

### Outcomes {12}

#### Primary outcome measure

Difference between the groups in the rate of participants with radiologically verified recurrence at 1 year after surgery. Recurrence is defined as any part of the stomach or other abdominal viscera located above the diaphragm at computed tomography (CT).

#### Secondary outcome measures

Between group differences in the following:

- Rate of radiologically verified recurrence at 3 years.
- Mean GSRS (gastrointestinal symptoms rating scale) component scores of reflux, abdominal pain, indigestion, diarrhea, and constipation at 3 months, 1 year, and 3 years postoperatively.
- Mean Short-Form 36 (SF-36) physical and mental component scores at 3 months, 1 year, and 3 years postoperatively.
- Mean Dakkak dysphagia scores at 3 months, 1 year, and 3 years postoperatively.
- Frequency of reflux disease-related symptoms (heartburn, reflux, chest pain, dysphagia of liquids and/or solids, odynophagia, globus sensation, respiratory symptoms, nightly cough, and odynophagia categorized on a four-level scale at 3 months, 1 year, and 3 years postoperatively).
- Rates and severity of complications at 30 days postoperatively. Complications will be classified according to Clavien-Dindo [34].
- Mean postoperative length of stay in days.
- Size and type of recurrence. Size will be measured in cm above the diaphragm. Type will be classified as types I–IV.

These metrics were chosen to assess both the objective and subjective effectiveness of the intervention as well as the safety. There is currently no core outcome set available that defines standardized outcome indicators for paraesophageal hernia repair, although it is in development [35]. The GSRS, SF-36, and Dakkak questionnaires are all validated instruments [36–38]. The reflux disease-related frequency questionnaire has been used in numerous previous studies from the same institution. Taken together, these instruments enable a comprehensive coverage of symptoms related to paraesophageal hernias.

### Participant timeline {13}

**Table:** 

	Enrolment	Allocation	Follow-up		Close-out
Timepoint	-t_1_	0	3 m	1 y	3 y
**Enrolment**					
Eligibility screen	X				
Informed consent	X				
Allocation		X			
**Interventions**					
Control		X			
Intervention		X			
**Assessments**					
QoL and symptom questionnaires	X		X	X	X
Computed tomography	X			X	X
Endoscopy	X				

### Sample size {14}

Previous studies have suggested recurrence rates after paraesophageal hernia repair ranging from 7 to 66% [15–20]. We have estimated the recurrence rate to be 30% in the control group and hypothesize that this can be reduced to 10% with the addition of gastropexy. With an α of 0.05 and a power of 80% (β = 0.2), we calculate a required sample size of 62 participants per arm, yielding a total of 124 evaluable participants. To account for an estimated 15% loss to follow-up, based upon previous studies with similar follow-up at the same institution, we aim to include 150 participants. The primary analysis will use multiple imputation under a missing-at-random assumption to include all randomized participants; this approach is expected to preserve the planned power under the anticipated missingness.

$$\begin{aligned}n&=\left[\sqrt{\frac{\left(0.3+0.1\right)}{2}\left(1-\frac{\left(0.3+0.1\right)}{2}\right)\left(1+\frac{1}{1}\right)}\ 1.96\ \right.\\&\left.+\ \sqrt{0.3\left(1-0.3\right)+\frac{0.1\left(1-0.1\right)}{1}}0.84\right]^{2}/{0.2}^{2}\approx 62\end{aligned}$$

### Recruitment {15}

This multicenter trial currently includes six centers, with additional centers being invited to participate. The annual volume of PEH repairs varies across the centers, but the majority perform more than 20 cases annually. Based on these volumes, we anticipate reaching our target of 150 participants included within the projected recruitment period of 2–3 years.

## Assignment of interventions: allocation

### Sequence generation {16a}

Participants will be assigned to either the control or intervention group with a 1:1 allocation ratio. A computer-generated randomization list in blocks of 10 for high-volume centers and six for low-volume centers will be used. Stratification will be performed by center only.

### Concealment mechanism {16b}

Allocation will be concealed using sealed, opaque, sequentially numbered envelopes. The envelopes will be opened in the operating theatre after induction of anesthesia, immediately prior to commencement of the procedure.

### Implementation {16c}

The randomization sequence will be generated centrally by the central study coordinator prior to trial initiation. Sealed envelopes will be prepared, shuffled independently by research nurses, and distributed to the participating sites. Participants will be enrolled locally by the treating surgeon or research nurse.

## Assignment of interventions: blinding

### Who will be blinded {17a}

The outcome assessors and participants will be blinded during the entire follow-up ending at 3 years after the surgical PEH repair. The operation report, with information on the specific type of repair performed, will not be included in the digital patient chart. Instead, a hard copy with information about the study group allocation will be kept in a sealed envelope, which is filed in a locked archive.

### Procedure for unblinding if needed {17b}

Unblinding is permissible if emergencies in the clinical management of the participant so require. Unblinding is performed by opening the sealed envelope, whereupon the procedural information is added to the medical records.

## Data collection and management

### Plans for assessment and collection of outcomes {18a}

Participants will undergo a study-specific low-dose CT scan of the upper abdomen and lower thoracic cavity at 1 and 3 years, with a permissible variation of ± 3 months from the specified time points. The CT protocol can be found in Supplement A. If a participant undergoes a clinically indicated CT scan that includes the upper abdomen and lower thoracic cavity within the specified time frame, the study-specific CT scan may be omitted. The CT scans will be reviewed by a specialized radiologist, blinded to the participants’ group allocation, to assess for recurrence.

Postoperative data including length of stay, complications, and reoperations will be collected by a research nurse or research fellow 30 days postoperatively. Severity of complications is graded according to the Clavien Dindo classification [34].

Patient-reported outcome measures (PROMs), including quality of life and the severity and frequency of symptoms, will be assessed at baseline, 3 months, 1 year, and 3 years. Health-related quality of life will be assessed with the Swedish version of the Short Form-36 (SF-36) questionnaire (RAND Corporation). SF-36 consists of 36 items grouped in eight domains, which can be summarized to mental and physical component scores [39–42].

The Gastrointestinal symptom rating scale (GSRS) measures symptoms of reflux, abdominal pain, indigestion, diarrhea, and constipation. The questionnaire consists of 15 items and uses a 7-point Likert scale, with 1 representing no symptoms and 7 representing severe symptoms. Scores are summarized for each of the five domains [38, 39].

The Dakkak dysphagia score assesses dysphagia. Participants report frequency (never, sometimes, or always) of dysphagia when eating nine types of solid, mashed, and liquid food. The generated score ranges from 0 to 45, where we will reverse the original scoring as done in previous studies, so that a higher score indicates more severe dysphagia [36, 37].

Additionally, a specific questionnaire will be used to assess the frequency of specific symptoms of heartburn, reflux, chest pain, dysphagia, globus sensation, respiratory symptoms, nightly cough, odynophagia as well as difficulty vomiting or belching.

The questionnaires will be made available to participants as hard copy or digital, depending on their preference. Participants will receive reminder letters by regular mail to encourage timely completion and submission of the forms.

### Plans to promote participant retention and complete follow-up {18b}

The questionnaires for postoperative follow-up are sent to the participants either by mail or electronically as preferred. If the forms are not completed and returned properly, a reminding letter will be sent by regular mail. If this is unsuccessful, a research nurse, research fellow, or surgeon will contact the participant by phone. The computed tomography at 1 and 3 years will, whenever possible, be performed at the participant’s local hospital to improve convenience for the participant.

### Data management {19}

Two methods of data entry will be used to maximize convenience for participants and research staff. Data will either be entered directly in an electronic case report form or on paper forms. Pseudonymized paper forms will be securely stored at each center until they have been fully completed, after which they will be sent to the research coordinator for manual entry into the database. All manually entered data will be double-checked for accuracy. Data will be stored in a pseudonymized database on protected servers with restricted access at Ersta hospital.

### Confidentiality {27}

Screening logs and identity logs will be kept in locked storage at each participating site. Pseudonymized data will be stored in a database at Ersta hospital.

### Plans for collection, laboratory evaluation, and storage of biological specimens for genetic or molecular analysis in this trial/future use {33}

No biological specimens will be collected.

## Statistical methods

### Statistical methods for primary and secondary outcomes {20a}

Analyses will be performed on an intention-to-treat basis. Descriptive statistics will be used to present overall and group-wise data.

The primary outcome will be analyzed using logistic regression with treatment group as the main independent variable, with pre-specified co-variates including age, BMI, and hiatal area.

Secondary categorical outcomes will be analyzed using logistic regression and continuous outcomes using linear regression. Continuous outcomes with repeated measurement will be analyzed using linear mixed-effect models.

Exploratory post hoc analyses will assess the association between surgeon experience, defined as the number of prior paraesophageal hernia repairs performed, and primary and secondary outcomes.

All analyses will be performed using R Statistical Software (version 4.4.1, 2024–06–14, R Core Team, Vienna, Austria).

### Interim analyses {21b}

No interim analyses are planned.

### Methods for additional analyses (e.g., subgroup analyses) {20b}

At present, no subgroup analyses are planned.

### Methods in analysis to handle protocol non-adherence and any statistical methods to handle missing data {20c}

With the randomization and intervention taking place in the operating room, we expect no protocol non-adherence. Prior to surgery, we will ensure that all preoperative questionnaires are completed, which should reduce the risk of missing baseline data. We will handle missing primary outcome data using multiple imputation by chained equations (MICE) under a missing-at-random assumption. The imputation model will include treatment group and pre-specified prognostic variables (including baseline characteristics and any available intermediate/follow-up measurements) to generate m imputed datasets; each dataset will be analyzed using the planned regression model for the dichotomous outcome (e.g., logistic regression), and estimates will be combined using Rubin’s rules.

### Plans to give access to the full protocol, participant level data, and statistical code {31c}

No public access will be granted to the full protocol, participant-level data, or statistical code.

## Oversight and monitoring

### Composition of the coordinating center and trial steering committee {5d}

The study will be coordinated by a research team at Ersta hospital including the principal investigator, surgeons, research fellows, and research nurses. The steering committee consists of the research team as well as surgeons from major participating sites. The steering committee is planned to have regular meetings approximately four times annually to monitor the progress of the study and to consider input from the data monitoring committee and safety committee.

### Composition of the data monitoring committee, its role and reporting structure {21a}

The trial has an independent data monitoring committee (DMC) consisting of three members. The DMC will monitor all data related to study participant inclusion, informed consent, and primary outcome. In addition, the DMC will monitor random samples from every fifth to tenth study participant to ensure adherence to protocol regarding the accuracy and completeness of case report forms, questionnaires, and the randomization process. The DMC will meet at least twice yearly and report findings to the trial steering committee. The full monitoring plan can be found in Supplement B.

### Adverse event reporting and harms {22}

All severe adverse events (SAE), defined as Clavien Dindo ≥ 3b, will be reported to a safety committee (SC) consisting of two independent members, both experienced senior surgeons. Data regarding SAEs provided to the SC by the study coordinator will be partially blinded, allowing treatment arms to be differentiated as group A and B, but allocation to the control arm or intervention arm will remain unknown to the SC. The SC may give recommendations on protocol alterations to the trial steering committee. The SAE report form can be found in Supplement C.

### Frequency and plans for auditing trial conduct {23}

The trial steering committee will evaluate trial conduct at scheduled meetings four times a year.

### Plans for communicating important protocol amendments to relevant parties (e.g., trial participants, ethical committees) {25}

Significant protocol amendments will be communicated without delay to all investigators as well as the national ethical review authority. Amendments that affect participants, including changes to follow up or data management, will be communicated to affected participants.

### Dissemination plans {31a}

The results of the trial will be published in a peer-reviewed medical journal targeting healthcare professionals and planned to be included in a doctoral thesis. All participants will be informed at the end of the study period whether they were allocated to the control or intervention arm.

## Discussion

Current guidelines usually recommend surgical treatment for patients with symptomatic PEH, but the optimal technical approach remains debated [7, 43–46]. Despite decades of experience with PEH repair, recurrence rates remain high, with reported rates even exceeding 50% [19].

The impact of tension created during hernia repair for recurrence rates is well documented in other types of hernia repair, including inguinal and ventral hernias [47]. It has been proposed that tension in PEH repair originates from two directions: axial tension related to repeated esophageal shortening and lateral tension caused by narrowing of the separated hiatal crura during the operation. In attempts to reduce tension and counteract the transdiaphragmatic pressure gradient promoting recurrence, various adjunct techniques have been evaluated.

Mesh reinforcement has been used with the aim to reduce the tensile forces at the crural suture line (mainly addressing the lateral tension). However, studies on the use of mesh adjacent to the esophagus in laparoscopic PEH repair have yielded conflicting results, with worrisome reports of severe mesh-related complications [48, 49]. Additionally, recent meta-analyses have shown similar long-term recurrence rates with or without mesh reinforcements, questioning its routine use [50–52]. An alternative to mesh reinforcement is to reduce the crural tension by adding a diaphragmatic relaxing incision. While retrospective studies have shown promising results, this has not been properly evaluated until quite recently [53]. Based on the lack of evidence behind the efficacy of mesh reinforcement, irrespective of the type of mesh used, the traditional crural repair should still be considered “standard of care,” especially when exploring other approaches to minimize the recurrence risk after PEH repair.

For addressing axial tension, procedures for esophageal lengthening such as wedge-fundectomy and traditional Collis gastroplasty have been proposed [2, 43, 54]. None of these techniques has so far been evaluated in a randomized clinical trial assessing recurrence following PEH repair. In clinical practice, several strategies to counteract axial forces have been employed, including various attempts to “anchor” the stomach and the reconstruction within the abdominal cavity. Placement of a gastrostomy tube has also been used to provide fixation of the anterior aspect of the stomach to the abdominal wall. Hernia reduction with gastropexy alone was described in the original studies from the early 1950 s [23, 55–57] but thereafter mostly abandoned, except as an alternative approach in high-risk patients [2]. While smaller series have reported promising results, up until quite recently, the use of gastropexy as an adjunct to a PEH repair in uncomplicated cases where the stomach and distal esophagus can be replaced to the abdomen without excessive tension has not been evaluated in a scientifically rigorous manner [2, 37, 58, 59]. However, in a recent randomized study, it was shown that an anterior gastropexy with two transfascial sutures significantly reduced the 1-year recurrence rate in patients undergoing PEH repair [60]. Notably, the majority of patients in this study did not receive an antireflux procedure, which is regarded as the established standard of care in PEH repair, supported by current evidence and recommended in clinical guidelines [9, 12, 13, 61]. Albeit this important study supports the relevance of gastropexy to reduce the risk of recurrence, a study is warranted to address the effect of gastropexy when added to a standard of care PEH repair.

In the current study protocol, we compare extensive gastropexy, with three fixation points, to no gastropexy at all in the control group. To minimize the risk of potential confounders due to technical differences, we have opted for a standardized PEH repair. While many surgeons may prefer at least some fixation of the stomach, evidence supporting the use of any type of gastropexy in PEH repair is only emerging. In addition, there are no data suggesting that several points for gastropexy reduce recurrence rates compared to fewer. We have therefore chosen to compare two “extremes”: gastropexy with a maximal fixation in the intervention group compared with no fixation at all in the control group. If our data show an advantage with the use of gastropexy, the optimal type of fixation needs to be addressed in future studies.

The primary endpoint in our study is radiologically verified recurrence at 1 year. Since many patients with recurrence of PEH after surgical repair report minor or no symptoms, it is important to assess whether observed differences between groups are clinically relevant [19]. To address this, our study incorporates PROMs evaluating both generic health-related quality of life and disease-specific symptoms. By combining objective and patient-reported outcomes, we aim to provide robust evidence to guide future clinical practice and improve long-term outcomes for patients undergoing PEH repair.

## Trial status

The trial is ongoing. Recruitment started 2023–06–27 and is expected to be completed by the end of 2026.

## Supplementary Information

Additional file 1: Supplement A. CT protocol.

Additional file 2: Supplement B. Monitoring plan.

Additional file 3: Supplement C. SC charter.

## Data Availability

Access to all data will be restricted to the study coordinator, principal investigator, research fellow, and statisticians.

## Abbreviations

CT: Computed tomography
GEJ: Gastroesophageal junction
PEH: Paraesophageal hernia
GERD: Gastroesophageal reflux disease
SF-36: Short Form-36
GSRS: Gastrointestinal symptom rating scale
HRM: High resolution manometry
ASA: American Society of Anesthesiologists (ASA)
PROM: Patient reported outcome measure
QoL: Quality of life
DMC: Data Monitoring Committee
SC: Safety Committee
RCT: Randomized controlled trial
SAE: Severe adverse event
