# Supplementary material for: Can gastropexy reduce the recurrence rate after paraesophageal hernia repair? A study protocol for a double blind, randomized, multicenter clinical trial
Source: Trials. 2026 Mar 16;27:281. doi: 10.1186/s13063-026-09578-7 (PMC13063770; doi:10.1186/s13063-026-09578-7)
Supplement: Supplementary file 1 — Additional file 1: Supplement A. CT protocol. [file 13063_2026_9578_MOESM1_ESM.pdf]

Supplement A

Studie PEH III K Lågdos***

SOS-kod: 84008

GENERELLT

**Användningsområde:**	• Forskningsprojekt kirurgkliniken. Patienter som skall undersökas ett och två år postop hiatushernia med DT nedre thorax-övre buk med intravenös kontrast.<br>• Lågdosprotokoll<br><br>*50ml Omnipaque 240 mgI/ml) blandas i 450ml liter vatten.*<br><br>Be pat dricka 1dl peroral kontrast på bordet precis innan undersökningen börjar

UNDERSÖKNING

1. FÖRBEREDELSER

**Patient:**	Allmänna frågor	
	• ID-kontroll<br>• Längd<br>• Vikt<br>• Graviditet	
**Information till patient:**	• Vikten av att ligga stilla<br>• Andningsinstruktioner	Be pat dricka 1dl peroral kontrast på bordet precis innan undersökningen börjar. Samma koncentration som till anastomoserna.<br>(*50ml Omnipaque (240 mgI/ml) blandas i 450ml liter vatten*.)

2. GENOMFÖRANDE

**Patientposition:**	Ryggläge<br>Fötterna först<br>Armarna uppåt
**Lodrät centrering:** |	Nedre thorax - Buk<br>Carina måste vara med
**Vågrät centrering:** —	Centrera patienten i mitten i höjdled
**Topogram:**	Frontal

------------------------------Serie 1: Övre Buk K Venfas

**Körning:**	Carina - Crista	
**Kriterier:**		
**Kontrast:**	Omnidos + lokal kontrasttabell Se länk<br>0,5gI/kg<br><br>Räkna maxdos i Omnivis:<br>- Män 100kg<br>- Kvinnor 80kg<br>Maxdos 180ml - ingen nedre minimum gräns	
**Bolus-tracking:**	Premonitoring:<br>ROI:	Mitt i levern<br>I bukaorta
**Injektionstid:**	30sek	
**Delay:**	Post Bolus Delay: 45sek	

3. EFTERARBETE

**Dokumentation:**	
**Bildhantering:**	Kontollera att bilder kommit över i PACS, sedan häng bilderna.

Supplement A

**Rekonstruktioner:**	Vid metall ska iMAR läggas till på rekonstruktionerna (ej tunna snitt) för att reducera metallartefakter.

Undersökningstyp (visningsprotokoll): DT Buk K

BILD PARAMETRAR								Kommentar
Serie	Plan	Snitt	Increment	Filter	Fönster	Fast Window	Safir	
1. Övre Buk K (Carina- Crista)	Ax	1	0,7	Br40	Abdomen	Off	3	Fix axial
	Ax<br>Cor<br>Sag	5	2,5	Br40	Abdomen	On	3	

4. PROGRAMPARAMETRAR

TEKNISKA PARAMETRAR			
	Topogram		
kV	110		
mA	15		
DT Scan			
	Serie 1<br>Övre Buk K		
Scanteknik	RoutineSpiralAdultAbdomen		
CARE Dose4D & CARE kV	Full		
CARE kV Grouping	Off		
CARE kV optimized for	Bone- Calcium		
CARE kV Quality ref. mAs@120 kV	100		
kV	120		
Quality ref. mAs	100		
CARE kV min. kV	100		
CARE kV max. kV	140		
Pitch	0,8		
Rot. Time (s)	0,5		
Absolut Start Delay (BT) (s)	10		
Post Bolus Delay (s)	45		
FAST ROI	Aorta		
Trigger Level @120kV (HU)	100		
Kollimering	32 x 0,7		
Safir	3		
