# Supplementary material for: Can gastropexy reduce the recurrence rate after paraesophageal hernia repair? A study protocol for a double blind, randomized, multicenter clinical trial
Source: Trials. 2026 Mar 16;27:281. doi: 10.1186/s13063-026-09578-7 (PMC13063770; doi:10.1186/s13063-026-09578-7)
Supplement: Supplementary file 2 — Additional file 2: Supplement B. Monitoring plan. [file 13063_2026_9578_MOESM2_ESM.pdf]

Monitoreringsplan och rapport

Studietitel:	**PEH III**
Studiekod (Erstas)	**244**
Ansvarig prövare<br>(den som signerar):	**Marcus Reuterwall Hansson**
Lokal monitor:	

Om dokumentet

- Dokumentenen används både som monitoreringsplan och monitoreringsrapport.
- Dokumentet är en mall. Vid monitorering sparas det som nytt dokument med dagens datum i studiemappen.
- I samband att studien godkänns i FoU-enhetens lokala Forskningsnämnd säkerställs att alla essentiella dokument finns.
- Ansvarig prövare bestämmer vilka parametrar som ska monitoreras och hur ofta monitorering ska ske under studiens gång.
- Parametrarna är indelade i olika studieprocesser. Vänstra delen av varje tabell är monitoreringsplanen, ansvarig prövare beslutar vilka parameterns som ska monitoreras och hur ofta monitorering ska ske. Den högra delen av varje process är monitoreringsrapporten som monitor fyller i. Under varje process finns utrymme för mer text om det inte får plats i kommentarsrutan.
- I slutet av dokumentet finns en åtgärdslista som fylls i av monitor.
- Innan dokumentet skrivs ut för signering uppdaterar monitor innehållsförteckningen.
- Signerat dokument sparas i studiemappen på G://
- Förkortningar
    - Forskningsperson = FP
    - SAE = Serious Adverse Avents

Innehållsförteckning

1.0 Omfattning av monitoreringsbesök/patienter ... 4

2.0 Studiens omfattning ... 4

3.0 Process - Inklusion och samtycke ... 5

4.0 Process - CRF/e-CRF och frågeformulär ... 6

5.0 Process - Randomisering ... 7

6.0 Process - Studiespecifika undersökningar ... 8

7.0 Process - SAE ... 9

8.0 Åtgärdslista ... 10

9.0 Signering ... 11

1.0 Omfattning av monitoreringsbesök/patienter

Monitorering sker 1 gång/halvår enligt monitoreringsplan. Omfattningen styrs av antalet inkluderade patienter.

Sista monitoreringen görs efter sista uppföljningen är gjord på sista patienten. Vid denna monitorering används mallen ”stängningsrapport”.

2.0 Studiens omfattning

Antal patienter som ska inkluderas:	Totalt 124 st	Multicenterstudie	Ja (endast Ersta monitoreras)	Randomiserad studie	Ja	Blindad studie	Ja

Röd kursiv text är som stöd, bör tas bort innan signering

Grön text ska/kan ersättas och göras svart innan signering

3.0 Process - Inklusion och samtycke

		Monitoreringsplan (fylls i av prövare)		Monitoreringsrapport (fylls i av monitor)	
**Punkt**	**Parameter**	**Ska monitoreras**	**Omfattning**	**Studie-ID på de monitorerade**	**Kommentar** *Kort kommentar av vikt alternativt hänvisa till en utförligare kommentar nedan.*
3.1	Finns signerat och journalfört samtycke?	Ja	Alla fp		
3.2	Är ID-logg korrekt ifylld?	Ja	Alla fp		
3.3	Är fp inkluderade i enlighet med inklusions- och exklusionskriterier?	Ja	Alla fp		
**Ev. utförligare kommentar till parameter ovan (ange nummer):**					
XX	*Lägg till fler rader vid behov*				

Röd kursiv text är som stöd, bör tas bort innan signering
Grön text ska/kan ersättas och göras svart innan signering

4.0 Process - CRF/e-CRF och frågeformulär

		**Monitoreringsplan (fylls i av prövare)**		**Monitoreringsrapport (fylls i av monitor)**	
**Punkt**	**Parameter**	**Ska monitoreras**	**Omfattning**	**Studie-ID på de monitorerade**	**Kommentar** *Kort kommentar av vikt alternativt hänvisa till en utförligare kommentar nedan.*
4.1	Är CRF komplett ifyllt?	Ja	Var 5-10:e fp		
4.2	Överensstämmer ASA i CRF med anestesi-bedömning i journal?	Ja	Var 5-10:e fp		
4.3	Är frågeformulär utskickade på rätt tidpunkt?	Ja	Var 5-10:e fp		

**Ev. utförligare kommentar till parameter ovan (ange nummer):**	
<< XX >>	*Lägg till fler rader vid behov*

5.0 Process - Randomisering

		Monitoreringsplan (fylls i av prövare)		Monitoreringsrapport (fylls i av monitor)	
**Punkt**	**Parameter**	**Ska monitoreras**	**Omfattning**	**Studie-ID på de monitorerade**	**Kommentar** *Kort kommentar av vikt alternativt hänvisa till en utförligare kommentar nedan.*
5.1	Är randomisering utförd enligt processbeskrivning	Ja	Var 5-10:e fp		
5.2	Är blindningen intakt?	Ja	Var 5-10:e fp		

**Ev. utförligare kommentar till parameter ovan (ange nummer):**	
<< XX >>	*Lägg till fler rader vid behov*

6.0 Process - Studiespecifika undersökningar

		Monitoreringsplan (fylls i av prövare)		Monitoreringsrapport (fylls i av monitor)	
**Punkt**	**Parameter**	**Ska monitoreras**	**Omfattning**	**Studie-ID på de monitorerade**	**Kommentar** *Kort kommentar av vikt alternativt hänvisa till en utförligare kommentar nedan.*
6.1	Är röntgen-undersökningarna utförda och vid rätt tidpunkt enligt protokoll?	Ja	Var 5-10:e fp		

**Ev. utförligare kommentar till parameter ovan (ange nummer):**	
<< XX >>	*Lägg till fler rader vid behov*

Röd kursiv text är som stöd, bör tas bort innan signering

Grön text ska/kan ersättas och göras svart innan signering

7.0 Process - SAE

		Monitoreringsplan (fylls i av prövare)		Monitoreringsrapport (fylls i av monitor)	
**Punkt**	**Parameter**	**Ska monitoreras**	**Omfattning**	**Studie-ID på de monitorerade**	**Kommentar** *Kort kommentar av vikt alternativt hänvisa till en utförligare kommentar nedan.*
7.1	Är SAE processen följd enligt protokoll?	Ja	Var 5-10:e fp		
**Ev. utförligare kommentar till parameter ovan (ange nummer):**					
<< XX >>	*Lägg till fler rader vid behov*				

Röd kursiv text är som stöd, bör tas bort innan signering

Grön text ska/kan ersättas och göras svart innan signering

8.0 Åtgärdslista

**Punkt** (enligt ovan)	**Datum** *(när upptäckt)*	**Fråga/Åtgärd** *Kopiera från kommentarer ovan, alternativt skriv fråga/ åtgärd*	**Ansvarig**	**Datum åtgärdat**
	ååååmmdd			

9.0 Signering

Monitor signering: ____

Namnförtydligande: ____

Datum: ____

Ansvarig prövare signering: ____

Namnförtydligande: ____

Datum: ____
