# Supplementary material for: Can gastropexy reduce the recurrence rate after paraesophageal hernia repair? A study protocol for a double blind, randomized, multicenter clinical trial
Source: Trials. 2026 Mar 16;27:281. doi: 10.1186/s13063-026-09578-7 (PMC13063770; doi:10.1186/s13063-026-09578-7)
Supplement: Supplementary file 3 — Additional file 3: Supplement C. SC charter. [file 13063_2026_9578_MOESM3_ESM.pdf]

Supplement C

Safety Committee (SC) Charter

Title: Can gastropexy reduce the recurrence rate after paraesophageal hernia repair? A double blind, randomized, multicenter clinical trial
Abreviation: PEH3
Principal investigator (PI): Anders Thorell
ClinicalTrials.gov identifier: NCT06107634

1. Introduction
This charter outlines the responsibilities and operational procedures for the independent SC of the PEH3 clinical trial. The charter defines the membership of the SC, the timing and purpose of its meetings and establishes confidentiality requirements, reporting procedures and monitoring guidelines.

2. Roles and Responsibilities
The primary responsibility of the SC is to safeguard the interests of current and future trial participants. The SC will review SAE reports and assess any significant safety concerns and, if necessary, make recommendations to the on modifications to the protocol or termination of the trial. The SC will act as an advisory body to the steering committee, which retains the final decision on the trial's course.

3. Membership
The SC will comprise of two independent upper GI surgeons with experience in clinical trials and surgical expertise relevant to paraesophageal hernia repair.

Membership in the SC is for the entire duration of the trial. If a member is unable to continue participation, the reason for this is documented and a new member with comparable qualifications are appointed by the steering committee .

4. Meetings
The SC will meet biannually (twice a year). Meetings may be held more frequently if significant safety concerns or trends in the data arise. The C, PI or steering committee may call to such meetings. An annual report summarizing safety and trial progress will be provided to the principal investigator and relevant regulatory bodies.

5. Meeting minutes
Meeting minutes must include at a minimum:

- Protocol number, study title
- Meeting date
- Agenda
- List of attendees, including SC members.
- Information reviewed, discussion and rationale for recommendations to the steering committee.
- A copy of the SC report.

6. Data Review Process

- **Access to Data**: The SC will have access to all SAE reports. The study coordinator will provide the primary and follow-up SAE reports to the SC. The SAE reports will be provided to the DMSC with pseudonymized allocation identifiers (A and B) to maintain partial masking.
- **SAE Review**: Each SAE report shall be reviewed, and the SC shall assess the relationship to the intervention and possible implications for trial safety.
- **Monitoring Review**: Aggregate data and trends shall be reviewed during biannual meetings to identify any significant safety signals or imbalances in SAE occurrence.

7. Reporting Protocol

- **Initial Reporting**: After each biannual meeting, the SC shall prepare a summary report detailing any safety recommendations, findings, and advice. This report will be directed to the study principal investigator and trial steering committee within two weeks of the meeting.
- **Urgent Recommendations**: If any critical safety issues are identified, the SC shall prepare a rapid report with recommended actions. This will be communicated within 24 hours to the study's principal investigator and trial steering committee.
- **Ongoing Monitoring**: The SC will also monitor any responses or actions taken following its recommendations and document these in subsequent meeting minutes.

8. Confidentiality and Conflict of Interest

SC members will sign a confidentiality agreement to protect the privacy of participants and integrity of the data. Members must disclose any potential conflicts of interest and recuse themselves from specific decisions where these may apply.

9. Amendments

This charter can be amended as needed during the study. Amendments will be documented with sequential version numbers and dates of revision. Rationale for amendment is recorded in meeting minutes. Revisions must be reviewed and accepted by the steering committee.

Supplement C

SC report

Date:

SAE:

**Number (SAE)**	
**Allocation identifiers**	
**Age**	
**Gender**	
**Severity** ***(Clavien Dindo)***	
IIIb	
IVa	
IVb	
V	
Beskrivning av händelsen	
Beskrivning av relevant häslohistorik	
Beskrivning av åtgärd	
**Outcomes:**	
Recovered	
Recovered with sequelae	
Not recovered	
Deceased	
Unknown	

Review

**Relationship to intervention:**	
Unrelated	
Possible	
Definite	

Advice:

Recommendations:

☐ The SC recommends that the study continues as planned
☐ The SC recommends alternations of the study protocol (see comments)
☐ The SC recommends termination of the study

Comments:
